# Incidence of Esophageal Cancer in the United States from 2001-2015: A United States Cancer Statistics Analysis of 50 States

**DOI:** 10.7759/cureus.3709

**Published:** 2018-12-10

**Authors:** Nicolas Patel, Bikramjit Benipal

**Affiliations:** 1 Internal Medicine, New York University School of Medicine, New York, USA; 2 Internal Medicine, Temple University, Philadelphia, USA

**Keywords:** esophageal cancer, uscs, incidence, epidemiology

## Abstract

Introduction

Esophageal cancer is one of the leading causes of death in males in the United States (US). Previous studies have analyzed incidence rates of esophageal cancer in the US using the data from the National Cancer Institute’s Surveillance, Epidemiology and End Results (SEER) program. However, given its limited patient population, certain groups and regions in the US are underrepresented. Our study utilizes the United States Cancer Statistics (USCS) database, which combines the SEER database with the Centers for Disease Control and Prevention's (CDC) National Program of Cancer Registries (NPCR) to cover all 50 states to examine the incidence of esophageal cancer.

Methods

The USCS registry was used to obtain data for esophageal cancer from 2001 to 2015. Incidence analysis was stratified based on sex, race, stage, histology, and US regional location/histology.

Results

The overall incidence of esophageal cancer from 2001-2015 was 4.7 per 100,000 people per year. Overall incidence rates were greatest for each stratification in males, blacks, distant disease, adenocarcinoma, and those in the Midwest with adenocarcinoma. Blacks, compared to other races, had the greatest statistically significant decrease in incidence between 2001-2015 (annual percent change (APC) -4.55). The incidence rate is also increasing the most rapidly in those with adenocarcinoma in the Northeast from 2011 to 2015 (APC 2.16).

Conclusion

In our study, we were able to determine the incidence of esophageal cancer using data from all 50 states in the US. Our findings of decreasing incidence in blacks and increasing incidence of adenocarcinoma in the Midwest and Northeast help elucidate the at-risk populations. Moreover, our findings help bring to light risk factors that may be contributing to the development of esophageal cancer and how diagnosis and surveillance can be improved based on these risk factors.

## Introduction

Esophageal cancer is the seventh leading cause of death in males in the United States (US) [1]. There were approximately 17,290 new cases of esophageal cancer and 15,850 deaths from esophageal cancer in 2018 [1]. The incidence varies based on a variety of risk factors and location within the US and other Western countries [2, 3]. Squamous cell carcinoma is the most common histology for esophageal cancer worldwide; however, in the US, adenocarcinoma is the most predominant histology [4]. Previous studies have used incidence rates for esophageal cancer in the US using the data from the National Cancer Institute’s (NCI) Surveillance, Epidemiology and End Results (SEER) program and have demonstrated an increased incidence of esophageal adenocarcinoma (EAC) and a decrease in the incidence of esophageal squamous cell carcinoma (ESCC) [2]. However, the SEER database is comprised of 18 cancer registries and only represents approximately 28% of the US population [5]. As a result, the SEER dataset underrepresents certain racial/ethnic groups and regions within the US. The United States Cancer Statistics (USCS) combines both the Centers for Disease Control and Prevention's (CDC) National Program of Cancer Registries (NPCR) and the SEER database to include data for all 50 states [6]. In this study, we performed a comprehensive analysis of esophageal cancer incidence rates in all 50 states between 2001 and 2015, including a novel analysis of differences in incidence among different regions of the US stratified by histology.

## Materials and methods

Incidence data for esophageal cancer between 2001 and 2015 was obtained from the USCS registry [7]. The USCS database provides the official federal statistics on cancer incidence and population data for all 50 states and the District of Columbia. Cases were stratified by tumor site: C15.0 cervical esophagus, C15.1 thoracic esophagus, C15.2 abdominal esophagus, C15.3 upper third of esophagus, C15.4 middle third of esophagus, C15.5 lower third of esophagus, C15.8 overlapping lesion of esophagus, and C15.9 esophagus, NOS. International Classification of Diseases (ICD) for Oncology, 3rd edition codes data was extracted for squamous cell carcinoma (8070/3) and adenocarcinoma (8140/3). Incidence data were stratified based on sex, race, stage, US regions (Northeast, Midwest, South, and West), and histology (squamous cell carcinoma and adenocarcinoma). Stage was subclassified as localized, regional, and distant disease. Incidence analysis used Tiwari et al., 2006 modifications for confidence interval [8]. The Joinpoint Regression Program (version 4.5.0.1, DigitCompass LLC, Maryland, USA) was used to generate incidence curves and calculate annual percentage change (APC) using the least squares method [9]. Incidence data are per 100,000 and were adjusted to the year 2000 US standard population. For all analysis, p<0.05 was considered statistically significant.

## Results

A total of 232,639 patients were included in the incidence analysis between 2001-2015 (Table 1). There was a total of 181,995 (78%) males and 50,644 (22%) females. There was a total of 231,592 patients with an identifiable race. Of those 201,260 (87%) were white, 24,878 (10.75%) were black, 1,170 (0.5%) were American Indian/Alaska Native, and 4,284 (1.85%) were Asian or Pacific Islander (API). There were 199,089 with an identifiable stage at the time of diagnosis (localized, regional, distant). Localized disease accounted for 47,693 (24%), 71,289 (36%) were regional, and 80,107 (40%) were distant. There was a total of 191,996 patients with a histology of squamous cell carcinoma or adenocarcinoma. Of those 64,088 (33%) were squamous cell carcinoma and 127,908 (67%) were adenocarcinoma. There was a total of 232,639 with a regional location within the United States (Northeast, Midwest, South, West) identified. Of those 49,656 (21%) were from the Northeast, 57,082 (25%) were from the Midwest, 82,028 (35%) were from the South, and 43,873 (19%) were from the West.

**Table 1:** Patient Characteristics

Patient Characteristics		
Gender (n=232,639)		
	Count	Percent
Male	181,995	78%
Female	50,644	22%
Race (n=231,592)		
	Count	Percent
White	201,260	87%
Black	24,878	10.75%
Asian or Pacific Islander	4,284	1.85%
American Indian/Alaska Native	1,170	0.50%
Stage (n=199,089)		
	Count	Percent
Localized	47,693	24%
Regional	71,289	36%
Distant	80,107	40%
Histology (n=191,996)		
	Count	Percent
Adenocarcinoma	127,908	67%
Squamous Cell Carcinoma	64,088	33%
Regions (232,639)		
	Count	Percent
Northeast	49,656	21%
Midwest	57,082	25%
South	82,028	35%
West	43,873	19%

**Figure 1:**
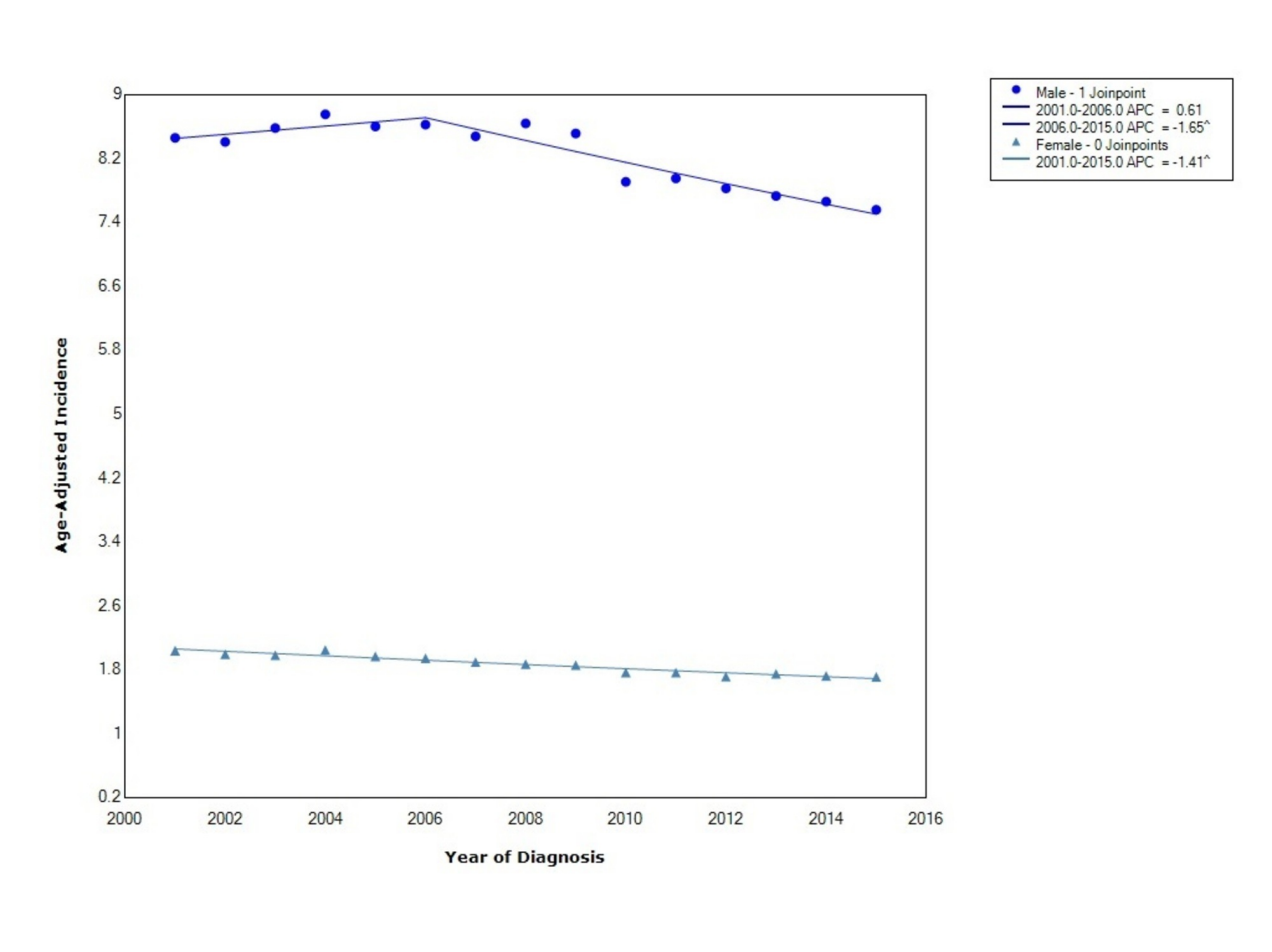
Incidence Rate, Sex APC: Annual Percent Change ^ Indicates that the APC is significantly different from zero at the alpha = 0.05 Age-Adjusted Incidences are per 100,000 and age adjusted to the 2000 United States standard population

The overall incidence of esophageal cancer from 2001-2015 was 4.7 per 100,000 people per year. Males had an incidence of 8.2 (95% CI 8.17-8.25), which was greater than females who had an incidence of 1.86 (95% CI 1.84-1.88). During this time, the incidence in females decreased with statistical significance with an APC of -1.41. In males, the APC initially was increasing from 2001 to 2006 with an APC of 0.61 and then started to decrease with statistical significance between 2006 to 2016 (Figure 1).

**Figure 2:**
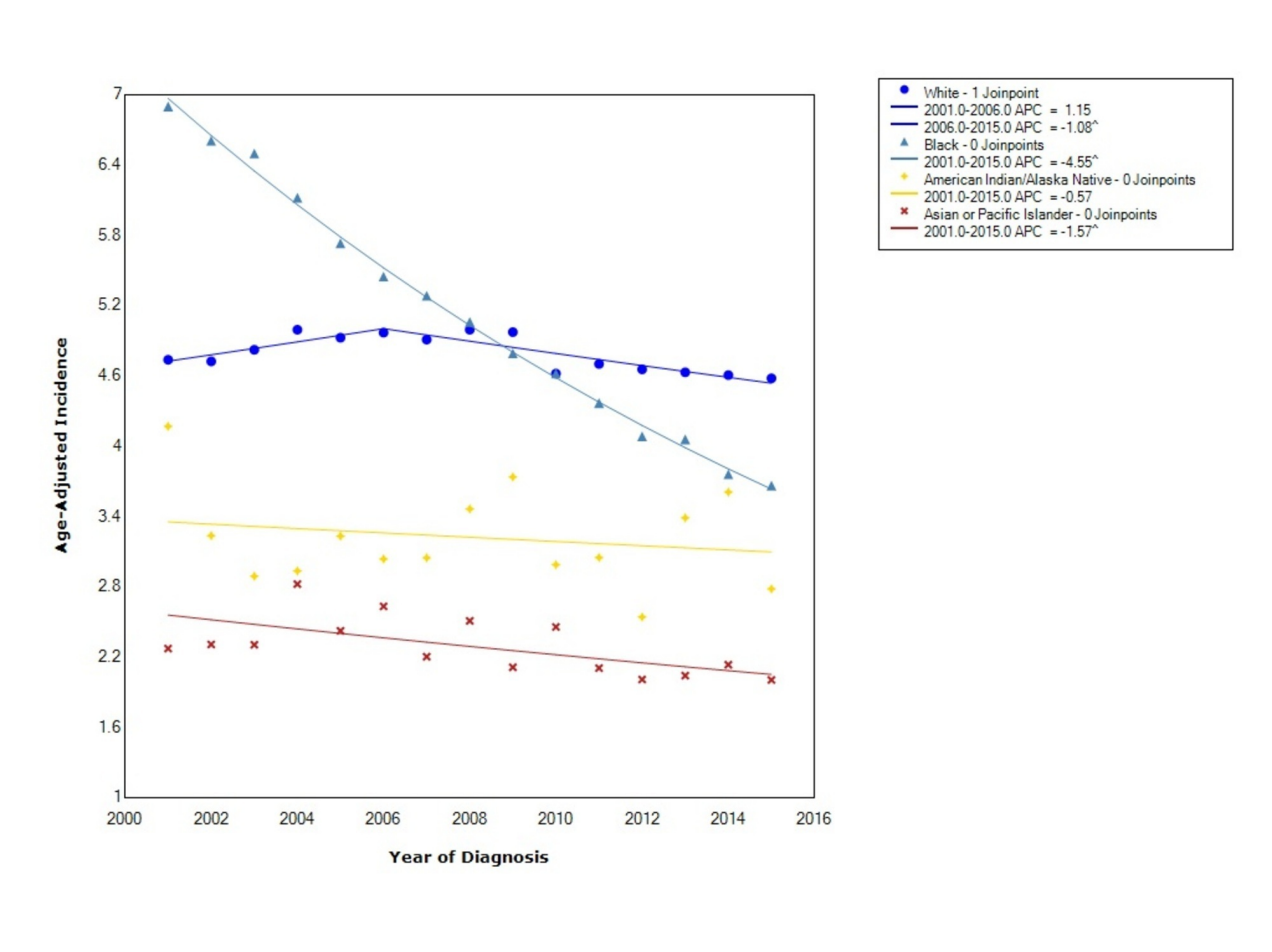
Incidence Rate, Race APC: Annual Percent Change ^ Indicates that the APC is significantly different from zero at the alpha = 0.05 Age-Adjusted Incidences are per 100,000 and age adjusted to the 2000 United States standard population

When stratified by race, esophageal cancer had the greatest overall incidence in blacks (4.99 95% CI 4.93-5.05), followed by whites (4.78 95% CI 4.76-4.80), American Indian/Alaska Native (3.18 95% CI 2.99-3.38), and lastly API (2.25 95% CI 2.18-2.32). Blacks had a statistically significant decreasing incidence between 2001-2015 (APC -4.55). For whites, the incidence increased insignificantly between 2001 and 2006 (APC 1.15) and then decreased with statistical significance between 2006 and 2015 (APC -1.08). The incidence in American Indian/Alaska Native decreased during this time (APC -0.57). APIs also had a decreasing incidence between 2001 and 2015 with a statistically significant APC of -1.57 (Figure 2).

**Figure 3:**
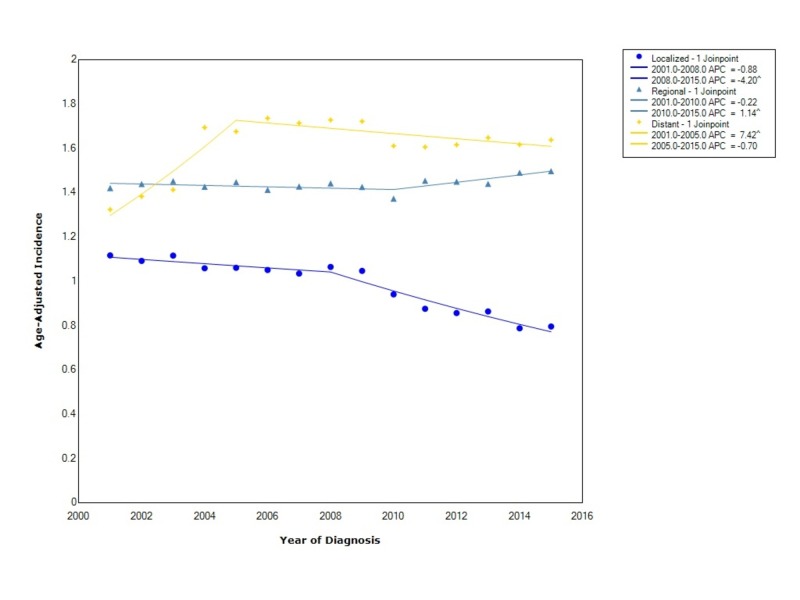
Incidence Rate, Stage APC: Annual Percent Change ^ Indicates that the APC is significantly different from zero at the alpha = 0.05 Age-Adjusted Incidences are per 100,000 and age adjusted to the 2000 United States standard population

When comparing esophageal cancer incidence stratified by stage at diagnosis, distant disease had the greatest overall incidence with a rate of 1.61 (95%CI 1.60-1.62), followed by regional disease with a rate of 1.44 (95% CI 1.43-1.45), and lastly localized disease with an incidence of 0.97 (95%CI 0.96-0.98). The incidence of localized disease decreased from 2001 to 2015; however, there was a significant decrease in incidence between 2008 and 2015 (APC -4.20). Regional disease initially was decreasing (APC -0.22); however, after 2010 the incidence started to increase with statistical significance (APC 1.14). Distant disease was increasing initially at a statistically significant rate (APC 7.42) from 2001 to 2005; however, after that time period, the incidence started to level off (APC -0.70) (Figure 3).

**Figure 4:**
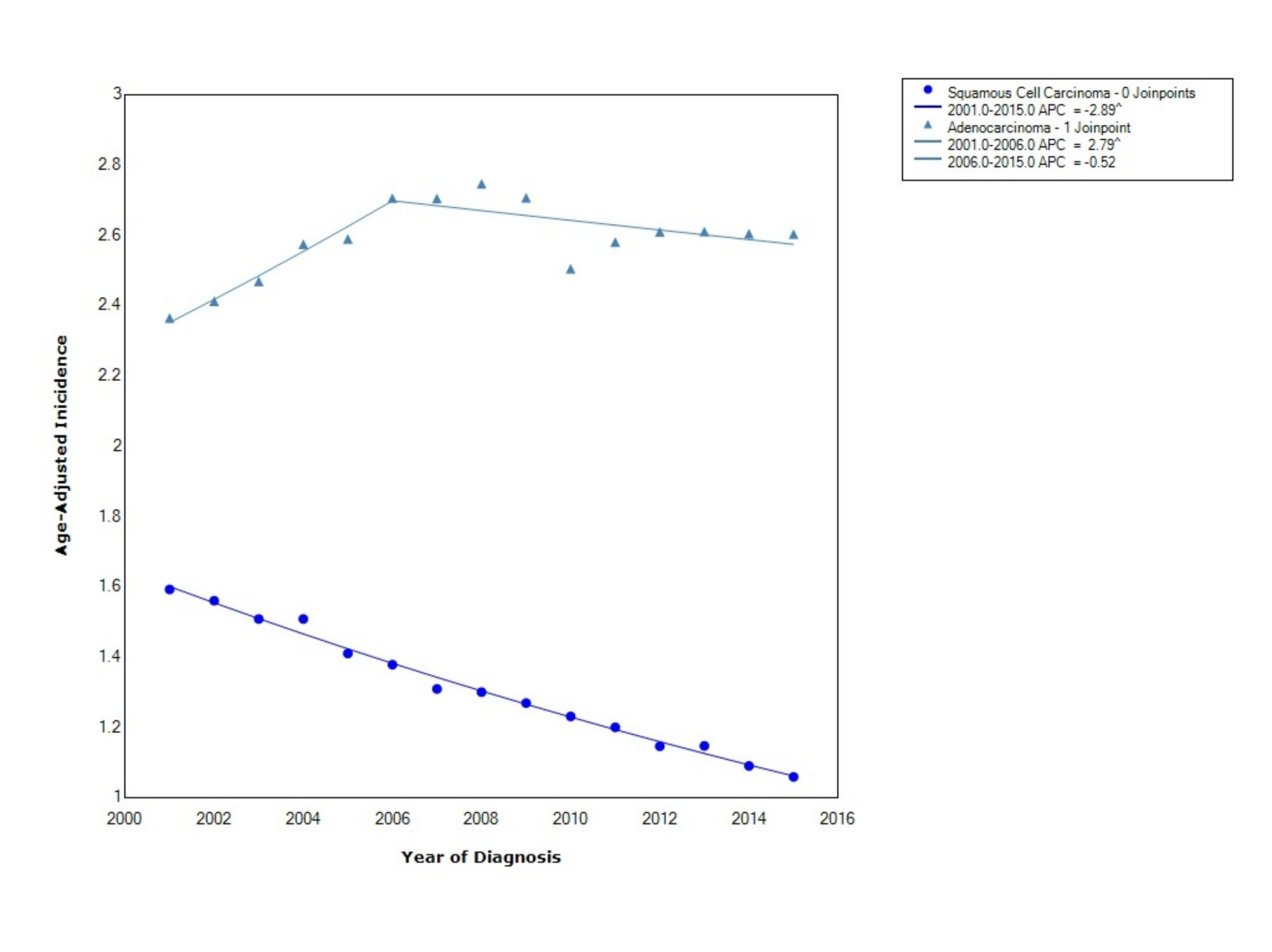
Incidence Rate, Histology APC: Annual Percent Change ^ Indicates that the APC is significantly different from zero at the alpha = 0.05 Age-Adjusted Incidences are per 100,000 and age adjusted to the 2000 United States standard population

Esophageal cancer incidence, when stratified by histology, shows that the overall incidence is greater for EAC (2.59 95% CI 2.57-2.61) than for ESCC (1.29 95% CI 1.28-1.30). The incidence of ESCC between 2001 and 2015 declined with statistical significance (APC -2.89). The incidence of EAC initially increased with statistical significance from 2001 to 2006 (APC 2.79); however, after that time period, the incidence started to decrease (APC -0.52) (Figure 4).

**Figure 5:**
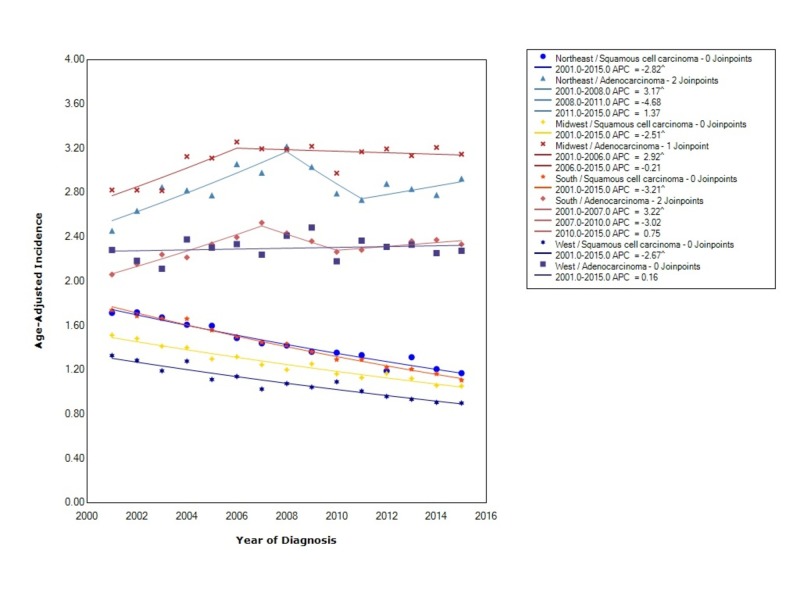
Incidence Rate, Region/Histology APC: Annual Percent Change ^ Indicates that the APC is significantly different from zero at the alpha = 0.05 Age-Adjusted Incidences are per 100,000 and age adjusted to the 2000 United States standard population

Incidence rates were also evaluated for US regions stratified by histology (ESCC and EAC). Compared to ESCC, EAC had the greatest incidence in all four regions. The incidence of adenocarcinoma was the greatest in the Midwest at 3.09 (95% CI 3.06-3.13), followed by the Northeast at 2.85 (95% CI 2.81-2.88), the South at 2.31 (95% CI 2.29-2.34), and lastly the West at 2.29 (95% CI 2.26-2.32). As seen in Figure 5, the incidence of squamous cell carcinoma was decreasing in all four regions. The incidence of adenocarcinoma varied in the four regions. In the West, the APC for EAC was 0.16. In the South, the incidence between 2001 and 2007 increased with statistical significance (APC 3.22). Between 2007 and 2010 the incidence decreased (APC -3.02) and then started increasing again thereafter (APC 0.75). In the Midwest, the incidence increased with statistical significance from 2001 to 2006 (APC 2.92) and then leveled off thereafter (APC -0.21). In the Northeast, the incidence initially increased with statistical significance from 2001 to 2008 (APC 3.17), then decreased until 2011 (APC -4.68), and then started to increase again (APC 1.37) (Figure 5).

## Discussion

Prior studies have shown that the incidence of esophageal adenocarcinoma is increasing and the incidence of esophageal squamous cell carcinoma is decreasing [2]. Important risk factors for esophageal adenocarcinoma include male sex, white race, gastroesophageal reflux disease, Barrett’s esophagus, obesity, tobacco, alcohol intake, and a diet low in fruits and vegetables [4]. Protective medications for esophageal adenocarcinoma include non-steroidal anti-inflammatory drugs (NSAIDs), proton pump inhibitor (PPIs) and statins in those with Barrett’s esophagus [4]. Risk factors for esophageal squamous cell carcinoma include black race, female sex in white individuals, smoking, alcohol, diets such as tea and coffee [4]. Prior studies have evaluated the incidence of esophageal cancer using databases that only include a limited representation of the US population. The purpose of our study was to evaluate the incidence of esophageal cancer using the USCS database, which includes all 50 US states and thus likely evaluates the cancer burden more accurately than other databases.

In our study, there was an overall 4.41:1 male to female incidence ratio. Incidence in females was decreasing the entire time from 2001 to 2015, whereas in males the incidence was initially increasing; however, after 2006 the incidence started to decrease at a rate greater than that in females. The overall incidence of EAC, when stratified by race, showed that blacks had the greatest overall incidence but that the incidence was also decreasing at the fastest rate. As above, an important risk factor for the development of ESCC is smoking. Over the years, blacks have had the steepest decline in smoking rates [10, 11]. Thus, our findings can possibly be explained by the decreasing incidence of ESCC due to declining smoking prevalence [10, 11].

In the study by Njei et al., they found that from 1973 to 2009 the incidence of distant disease was the greatest, but that the diagnosis of localized disease was on the rise [2]. Our findings, looking at all 50 states, show that the incidence of distant disease was similarly the greatest overall but that the incidence of localized disease since 2008 has been declining at the greatest rate. Moreover, the diagnosis of regional disease is on the rise. This is a concerning finding as esophageal cancer is being diagnosed at a later stage than localized and implies a need for better screening and surveillance guidelines for those with known risk factors for esophageal cancer.

Njei et al., also found that there was a rising incidence of esophageal adenocarcinoma compared to squamous cell carcinoma (SCC) [2]. Our findings show that the incidence of ESCC has continued to decline, and unlike the findings of Njei et al., the incidence of EAC after a period of rising has begun to decline as well. The decline in the incidence of ESCC can be explained by a reduction in tobacco and alcohol consumption [2]. The declining incidence of esophageal adenocarcinoma shows that there may be improvements in the diagnosis and treatment of underlying risk factors for EAC such as diet, tobacco, and gastroesophageal reflux disease than before.

In this study, we also took a novel approach to evaluate esophageal cancer incidence based on regional location within the US and histology. We found that the incidence of ESCC was decreasing in all regions. When comparing the incidence of EAC based on regional location, we found that the overall incidence of EAC was greatest in the Midwest but currently increasing at the fastest rate in the Northeast. The prevalence of obesity is greater in the Northeast and Midwest compared to other regions and thus it may be a significant risk factor for the development of EAC in these regions [12]. Moreover, these findings may be related to unidentified regional variations in risk factors that are more prevalent in certain areas compared to others [12].

There were a few limitations to our study. The USCS dataset includes a wealth of data for all 50 states; however, the ability to stratify data based on specific risk factors, such as smoking history or alcohol intake, is not available and thus we were not able to make inferences on specific influences for the trends we found. Moreover, we analyzed data at a regional level and thus risk factors on a state level would require further analysis. The strengths of our study include the use of the USCS database that covers all 50 states and the fact that it thus estimates incidence rates more precisely than other databases that do not account for all populations.

## Conclusions

In our study evaluating the incidence of esophageal cancer in all 50 states, we found that the overall incidence is greatest in males, blacks, distant disease, EAC, and those in the Midwest with EAC. We also found that for race, black individuals have the fastest decline in incidence and that for the stage at diagnosis, regional disease is on the rise. Moreover, although the overall incidence of EAC has started to decline in the US, there is still a significant overall incidence of EAC in the Midwest and a rising incidence in the Northeast, which may be related to higher rates of obesity in these areas. Our study is the first to evaluate esophageal cancer incidence in all 50 states and provides important trends and risk factors for the development of this cancer. Ultimately, we will need to use this data to improve surveillance guidelines for at-risk populations.
